# Precoded spatial multiplexing MIMO system with spatial component interleaver

**DOI:** 10.1186/s13638-016-0558-8

**Published:** 2016-02-29

**Authors:** Xiang Gao, Zhanji Wu

**Affiliations:** School of Information and Communication Engineering, Beijing University of Posts & Telecommunications, Xitucheng Road 10, Beijing, 100876 China

**Keywords:** Multiple-input multiple-output (MIMO), Bit-interleaved coded modulation (BICM), Signal space diversity (SSD), Spatial multiplexing, Precoding, Rayleigh channels

## Abstract

In this paper, the performance of precoded bit-interleaved coded modulation (BICM) spatial multiplexing multiple-input multiple-output (MIMO) system with spatial component interleaver is investigated. For the ideal precoded spatial multiplexing MIMO system with spatial component interleaver based on singular value decomposition (SVD) of the MIMO channel, the average pairwise error probability (PEP) of coded bits is derived. Based on the PEP analysis, the optimum spatial Q-component interleaver design criterion is provided to achieve the minimum error probability. For the limited feedback precoded proposed scheme with linear zero forcing (ZF) receiver, in order to minimize a bound on the average probability of a symbol vector error, a novel effective signal-to-noise ratio (SNR)-based precoding matrix selection criterion and a simplified criterion are proposed. Based on the average mutual information (AMI)-maximization criterion, the optimal constellation rotation angles are investigated. Simulation results indicate that the optimized spatial multiplexing MIMO system with spatial component interleaver can achieve significant performance advantages compared to the conventional spatial multiplexing MIMO system.

## Introduction

Coded modulation is one of the pivotal techniques of wireless communications. High spectral efficiency and link reliability are always the challenges and goals of wireless communication systems. Nowadays, bit-interleaved coded modulation (BICM) multiple-input multiple-output (MIMO) technology has become one of the fundamental technologies and has been widely used in current wireless communication standards, such as IEEE 802.11ac, 3GPP LTE [1–5].

For the MIMO system, spatial multiplexing is an effective method to multiply channel capacity and spectral efficiency. A high data rate signal stream is mapped onto multiple layers and each low-rate substream is transmitted simultaneously [6–8]. Unfortunately, spatial multiplexing is sensitive to ill-conditioning of the channel matrix. Besides, MIMO can also be implemented to obtain the diversity gain. Precoding and space-time coding (STC) are the most commonly used technologies. The basic idea of precoding is to use some form of channel state information (CSI) at the transmitter and receiver to customize the transmitted signal to the eigenstructure of the matrix channel [8–13]. The precoding based on singular value decomposition (SVD) with full CSI is known to achieve the MIMO channel capacity. After the SVD of channel matrix, the transmit precoding and receiver shaping transform the MIMO channel into *M* independent single-input single-output (SISO) channels, where *M* is the number of the transmit layers [8]. The most serious drawback of SVD precoding is the requirement of complete CSI at both the transmitter and receiver. To reduce the data for the feedback channel, limited feedback (LF) precoding schemes [13–17] were proposed, where a finite set of pre-determined unitary precoding matrices, referred to as the unitary codebook, are known to both the transmitter and the receiver. The receiver only needs to feedback the index of the precoding matrix as a function of the current CSI over a limited feedback channel. This practical approach significantly reduces the feedback overhead of MIMO systems.

However, a substantial tradeoff between spatial diversity and spatial multiplexing gains exists in a MIMO system [18–22]. How to get the maximum diversity gain and multiplexing gain at the same time is the main concern. Space-time bit-interleaved coded modulation (ST-BICM) is a full-rate space-time code to obtain high diversity and coding gain on MIMO channel [23, 24]. Through the serial concatenation of a channel encoder, an optimized bit interleaver, and a space-time signal constellation mapper, the diversity order and the coding gain depend on the Hamming distance of certain coded bit sub-sequences. In order to exploit the maximum diversity gain, the joint design of the code, the bit interleaver, and the space-time constellation mapper has great importance. A similar scheme named bit-interleaved coded multiple beamforming (BICMB) is proposed in [25]. It showed that for any convolutional code and any spatial demultiplexer, the maximum achievable diversity order is related with the product of the code rate and the number of streams. In fact, ST-BICM and BICMB can be viewed as the space-time extension of the BICM concept. The optimal performance is based on the ideal interleaving condition or a optimized interleaver which enables a global optimization taking into account channel coding. Therefore, it put forward a very high request to the design of bit interleaver to achieve the ideal interleaving condition which is highly correlated with the channel coding and MIMO configuration.

Signal space diversity (SSD) is an effective method to achieve the diversity gain and improve the error performance in fading channel without additional power or bandwidth [26]. The 2-dimensional SSD can be implemented by the combination of constellation rotation and component interleaving. Application of SSD in SISO systems has been widely studied [27–33]. In contrast, the research of SSD technique in coded MIMO systems remains undeveloped. Combining SSD with MIMO system is discussed in [34–40]. A coded MIMO scheme for block-fading channels that consists of a channel code and a space-time code based on SSD was proposed in [34]. In [35], the authors extended the SSD scheme to V-BLAST MIMO systems in order to achieve the maximum diversity gain without additional power or bandwidth consumption. The spatial multiplexing MIMO-OFDM system with a random swapping coordinate interleaver was proposed in [36, 37]. We also proposed a DFT-S-OFDM scheme based on SSD and applied SSD to the WLAN [38, 39]. Nevertheless, the proposed schemes and their optimization methods in current literatures still have many limitations and shortcomings. For instance,

- Most of the proposed MIMO systems with SSD in existing literature are merely an extension of SISO-SSD system, such as the proposed schemes in [36, 37]. It is simply a combination of spatial multiplexing MIMO and time domain component interleaving on each layer. In order to achieve the diversity gain, the variability of fading on time domain needs to be guaranteed. However, the spatial dimension provided by MIMO channel is unconsidered.
- The optimal rotation angles in the current research mainly based on the optimization of uncoded system [34–36], such as the maximum product distance criterion introduced in [26], or based on the extensive computer simulation [38, 39]. As for the coded MIMO scheme, actual operating signal-to-noise ratio (SNR) is quite low that it invalidates the results. Hence, the angle values that depend on the maximum product distance do not lead to the best error performance for the coded modulation MIMO schemes.
- For the coded MIMO system with SSD, the theoretical performance analysis is very difficult. Compared with SISO system, coded MIMO system with SSD lacks the theoretical analysis of the performance. The performance of proposed MIMO-SSD systems in [34–39] are evaluated by simulations.
- For the coded spatial multiplexing MIMO system, existing literatures mainly focus on the application and optimization of SSD with open-loop MIMO or SVD-precoded close-loop MIMO. Few studies on the SSD technology concentrated on limited feedback precoding.

In [40], a novel coordinate-interleaved spatial multiplexing (CISM) scheme based on SVD precoding is proposed. CISM diagonalizes the MIMO channel and interleaves the coordinates of the transmitted symbols over different layers to improve the diversity gains of the weaker eigenmodes resulting in a significant gain in the overall diversity. Compared with the ST-BICM and BICMB, the overall error rate performance can be significantly improved just by a simple component level interleaving with very short interleaving depth instead of the joint optimization of channel coding and specially designed bit level interleaver. Based on the symbol error probability analysis, the optimal constellation rotation angle and diversity-multiplexing tradeoff are analyzed. It enlightens us to utilize the natural spatial features of MIMO and extend the SSD to the spatial dimension. In this paper, channel encoding is added to the CISM scheme and a similar precoded BICM spatial multiplexing MIMO system with spatial component interleaver among transmit layers is studied. We focus on the optimization of the system performance with channel coding. The main contributions of this paper are as follows.

- The optimum spatial Q-component interleaver design criterion for the proposed CISM scheme with SVD precoding is discussed.
  An efficient spatial Q-component interleaver is proposed in [40] (Eq. (5)). It is one of the important foundation of SEP analysis and strongly associated with the conclusion in [40]. However, the proposed interleaver lacks theoretical basis. In this paper, we first investigate the performance of an ideal SVD-precoded BICM CISM scheme. Based on the analysis of average PEP of coded bits, the optimum spatial Q-component interleaver design criterion is provided to attain the minimum error probability. We proved that the optimum spatial Q-component interleaver is exactly the interleaver proposed in [40] and consummate the theoretical analysis of [40].
- The optimal precoding matrix selection criterion that is suitable for the proposed MIMO scheme with LF precoding is discussed.
  In [40], the CISM scheme with SVD precoding is studied. In this paper, the performance of the proposed coded CISM scheme with practical LF precoding is analyzed. Based on the linear zero forcing (ZF) receiver, in order to minimize a bound on the average probability of a symbol vector error, a novel effective SNR-based precoding matrix selection criterion and a simplified criterion that are suitable for the proposed MIMO scheme are proposed.
- The optimal constellation rotation angle of the proposed coded MIMO scheme is investigated.
  In [40], the optimal rotation angle obtained by SEP analysis is only applicable to the uncoded CISM scheme with SVD precoding. For the coded MIMO SSD system, the optimal angle depends on many factors, i.e., the number of antennas, the number of transmit layers, code rate, and modulation, which are usually ignored in present papers. Average mutual information (AMI) is an effective means to reflect the system performance and has been widely used in system optimization [32, 41–43]. A non-asymptotic space-time block code (STBC) design criterion based on the bitwise AMI maximization at a specific target SNR is proposed in [43]. It establishes the relation curve between the optimal design parameter *θ* and SNR. Therefore, the operating SNR should be determined before selecting the optimal parameter. In this paper, the BICM-AMI is used to search for the optimal constellation rotation angle. Based on the AMI-maximization criterion, the relationship between the system achievable rate and the optimal angle is established. It provides a direct reference to choose the optimal angle without determination of SNR.

Simulation results verify the theoretical analysis and show that the optimized spatial multiplexing MIMO system with spatial component interleaver can achieve significant performance advantages.

Throughout this paper, we use bold letters to represent vectors or matrices. (·)^*T*^ and (·)^*H*^ represent transposition and conjugate transposition. tr(·) represents trace of a matrix. SNR = *E*_r_/*N*_0_, where *E*_r_ denotes the average symbol energy per receive antenna and *N*_0_=2*σ*^2^ denotes the variance of the complex Gaussian noise. Extensive literature has proven that Gray labeling is optimal for BICM system [44]. Therefore, Gray labeling is employed in this paper.

The paper is organized as follows. The spatial multiplexing MIMO scheme with spatial Q-interleaver is proposed in Section 2. Performance analysis about the proposed scheme with optimum SVD precoding is given in Section 3. The limited feedback unitary precoding for the proposed scheme is discussed in Section 4. Based on the AMI analysis, the optimal rotation angles for proposed systems are presented in Section 5. Simulation results are presented in Section 6 on Rayleigh fading channels. Concluding remarks are offered in Section 7.

## System model

**Fig. 1:**
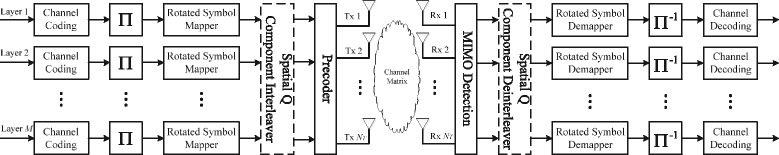
System model for spatial multiplexing MIMO with spatial component interleaver

The general model of *M*-layer spatial multiplexing MIMO system with spatial component interleaver is illustrated in Fig. 1. The MIMO system is equipped with *N*_*T*_ transmit antennas and *N*_*R*_ receive antennas, *M*≤ min{*N*_*R*_,*N*_*T*_}. Uniform power allocation for each layer is considered in this paper. As can be seen from Fig. 1, the CISM scheme introduced in [40] is an uncoded case of the proposed scheme with SVD precoding. In the transmitter, each of the *M* layers is encoded and modulated independently. For the *l*th (1≤*l*≤*M*) layer, *K* information bits are encoded and interleaved to yield the *N* length coded bit sequence, where the code rate $R = \frac {K}{N}$. Afterwards, *m*-tuple-coded bits are mapped to a complex symbol ${{x_{k}^{l}}} = {{x_{k}^{l}}}(I) + {\mathrm {j}} \cdot {{x_{k}^{l}}}(Q)$ which is chosen from a 2^*m*^-ary rotated QAM constellation set $\chi = \left \{\hat {x}_{1},\hat {x}_{2},...,\hat {x}_{2^{m}}\right \}$ according to the optimal angle. A spatial Q-component interleaver is applied for the Q-components of *M* symbols at the same instant according to the interleaving criterion *f*(*l*):

(1) $$ {s}_{k}^{n}(Q) = {x}_{k}^{l}(Q), n=f(l),  $$

where ${s}_{k}^{n}$ denotes the *k*th symbol at the *n*th (*n*∈[ 1,*M*]) layer after the spatial Q-component interleaver. Obviously, I-components keep the same layer-order as before, and just Q-components change the layer-order.

The precoder in the transmitter takes the symbol vector $\textbf {s} = {\left (s_{k}^{1},s_{k}^{2},\ldots,s_{k}^{M} \right)^{T}}$ as input and generates a vector ${\textbf {v}}=\left ({v_{k}^{1}},v_{k}^{2},\ldots,{v}_{k}^{N_{T}}\right)^{T}$ to be mapped onto *N*_*T*_ transmit antenna ports, where ${v}_{k}^{p}$ represents the *k*th symbol on the transmit antenna port *p*. Assuming perfect timing, synchronization, and sampling, the received signal vector ${\mathbf {r}}=\left [r_{k}^{1},{r_{k}^{2}},\ldots,r_{k}^{N_{R}}\right ]^{T}$ in the receiver can be expressed as follows.

(2) $$ \mathbf{r}=\mathbf{H}\cdot \mathbf{F}\cdot \mathbf{s}+\mathbf{n},  $$

where **H** is the *N*_*R*_×*N*_*T*_ MIMO channel matrix. In this paper, the entries of **H** is supposed to be independent and identically distributed (i.i.d.) according to ${\mathcal {CN}}(0,1)$. **F** is the precoding matrix. $\mathbf {n}=\left [n_{k}^{1},{n_{k}^{2}},\ldots,n_{k}^{N_{R}}\right ]^{T}$ denotes a column vector of *N*_*R*_ complex Gaussian random variables with mean zero and variance ${\sigma ^{2}} = \frac {{{N_{0}}}}{2}$. Assuming the perfect CSI, after the MIMO detection and corresponding spatial Q-component deinterleaving, the received symbol on *l*th layer is reconstructed as ${y_{k}^{l}}={{y_{k}^{l}}(I)} + {\mathrm {j}} \cdot {{y_{k}^{l}}(Q)}$ that corresponds to ${x_{k}^{l}}$ in the transmitter. A serial concatenation of a soft-in-soft-out rotated symbol demapper and a channel decoder is employed on each layer to approach the maximum likelihood (ML) receiver performance. For the *l*th layer, *l*∈[1,*M*], assume that the code sequence **c**_*l*_ is transmitted and $\hat {\mathbf {c}}_{l}$ is detected. The *j*th coded bits ${c_{l}^{j}}$ is mapped to the *i*th bit of *k*th symbol ${x_{k}^{l}}$. The soft demapper calculates the log-likelihood ratio (LLR) as follows.

(3) $$ L_{{c_{l}^{j}}} \triangleq = \ln \frac{P\left({c_{l}^{i,k} = 0|{y_{k}^{l}}} \right)}{P\left({c_{l}^{i,k} = 1|{y_{k}^{l}}} \right)}= \ln \frac{\sum\limits_{{{\hat x}} \in {\chi}_{i}^{0}} {P\left({{y}}_{k}^{l} |{{x}}_{k}^{l} = {\hat{x}}\right)}}{\sum\limits_{{{\hat x}} \in {\chi}_{i}^{1}} {P\left({{y}}_{k}^{l} |{{x}}_{k}^{l} = {\hat{x}}\right)}},  $$

where ${\chi }_{i}^{\alpha }$ denotes the subset of all signals *x*∈**χ** whose label has the value *α*∈{0,1} in position *i*. According to the LLRs, the information bits are decoded via channel decoder.

## Performance analysis of the proposed scheme with SVD precoding

Based on knowledge of the full CSI at the transmitter, the ideal precoding matrix can be obtained by SVD of the MIMO channel [6, 8]. The well-known SVD transceiver divides MIMO channel into parallel independent SISO channels and transmits multiple streams over the resulting eigenmodes. According to the SVD criterion, the MIMO channel matrix **H** is decomposed as

(4) $$ {\textbf{H}} = {\textbf{U}}{\mathbf{\Lambda}}{\textbf{V}}^{H},  $$

where the *N*_*R*_×*N*_*R*_ matrix **U** and the *N*_*T*_×*N*_*T*_ matrix **V** are unitary matrices. **Λ** is a *N*_*R*_×*N*_*T*_ diagonal matrix with singular values ${\lambda _{i}} \in {{\mathbb {R}}^ + }$ of **H** on the main diagonal in decreasing order. We denote $\bar {\mathbf {U}}_{\left [M \right ]}$ and $\bar {\mathbf {V}}_{\left [ M \right ]}$ as the first *M* column vectors of **U** and **V**, respectively. As introduced in [13, 40, 45], for the *M* layer spatial multiplexing MIMO system, the precoding and detection process can be expressed as linear transformations ${\textbf {z}}=\bar {\mathbf {U}}_{\left [ M \right ]}^{H} {\mathbf {H}} \bar {\mathbf {V}}_{\left [ M \right ]} \mathbf {s}+\bar {\mathbf {U}}_{\left [ M \right ]}^{H} {\mathbf {n}}$.

After the spatial Q-component deinterleaving, for the *k*th received symbol on *l*th layer, the fading coefficients of I-component and Q-component are different that can be expressed as

(5) $$\begin{array}{*{20}l} {y_{k}^{l}} (I) &= \lambda_{l} {x_{k}^{l}} (I) + {n_{k}^{l}} (I) \\ {y_{k}^{l}} (Q) &= \lambda_{f(l)} {x_{k}^{l}} (Q) + {n_{k}^{l}} (Q), \end{array} $$

where ${{n_{k}^{l}}={n_{k}^{l}} (I) + {\mathrm {j}} \cdot {{n_{k}^{l}} (Q)}}$ are complex Gaussian random variables with mean zero and variance per component ${\sigma ^{2}} = \frac {{{N_{0}}}}{2}$. For the *l*th layer, the PEP of **c**_*l*_ and $\hat {\mathbf {c}}_{l}$ can be expressed as

(6) $$ \begin{aligned} P\left({{\textbf{c}_{l}} \to {\hat{\mathbf{c}}_{l}}|\textbf{H}} \right) = P\left({\sum\limits_{j} {\mathop {\min }\limits_{x \in \chi_{i}^{{c_{l}^{\,j}}}}} \left({{{\left| {{y_{k}^{l}}(I) - {\lambda_{l}}x(I)} \right|}^{2}} + {{\left| {{y_{k}^{l}}(Q) - {\lambda_{f(l)}}x(Q)} \right|}^{2}}} \right) \ge} \right.\\ \left. {\sum\limits_{j} {\mathop {\min }\limits_{x \in \chi_{i}^{{\hat{c}_{l}^{\,j}}}}} \left({{{\left| {{y_{k}^{l}}(I) - {\lambda_{l}}x(I)} \right|}^{2}} + {{\left| {{y_{k}^{l}}(Q) - {\lambda_{f(l)}}x(Q)} \right|}^{2}}} \right)} \right). \end{aligned}  $$

In this paper, convolutional code is employed as the channel code. The Hamming distance between **c**_*l*_ and $\hat {\mathbf {c}}_{l}$ is at least *d*_free_. Without loss of generality, we assume $d\left ({\textbf {c}_{l}},{\hat {\mathbf {c}}_{l}} \right) = {d_{{\text {free}}}}$. Thus, the elements in $\chi _{i}^{{c_{l}^{\,j}}}$ and $\chi _{i}^{\hat {c}_{l}^{\,j}}$ are equal for all *j* except for *d*_free_ distinct values of *j*.

For the *d*_*free*_ bits, we define $\hat {x}_{k}^{l} = \arg \mathop {\min }\limits _{x \in \mathbf {\chi }_{i}^{\hat {c}_{l}^{\,j}}}\left ({{{\left | {{y_{k}^{l}}(I) - {\lambda _{l}}x(I)} \right |}^{2}} + {{\left | {{y_{k}^{l}}(Q) - {\lambda _{f(l)}}x(Q)} \right |}^{2}}} \right)$. By the same method introduced in [46], the PEP can be upper-bounded by

(7) $$ \begin{aligned} & P\left({{\textbf{c}_{l}} \to {\hat{\mathbf{c}}_{l}}|\textbf{H}} \right) \le P\left({\sum\limits_{j,{d_{{\text{free}}}}} {\left({{{\left| {{y_{k}^{l}}(I) - {\lambda_{l}}{x_{k}^{l}}(I)} \right|}^{2}} + {{\left| {{y_{k}^{l}}(Q) - {\lambda_{f(l)}}{x_{k}^{l}}(Q)} \right|}^{2}}} \right)} \ge} \right.\\ &\qquad \qquad \qquad \left. {\sum\limits_{j,{d_{{\text{free}}}}} {\left({{{\left| {{y_{k}^{l}}(I) - {\lambda_{l}}\hat{x}_{k}^{l}(I)} \right|}^{2}} + {{\left| {{y_{k}^{l}}(Q) - {\lambda_{f(l)}}\hat{x}_{k}^{l}}(Q) \right|}^{2}}} \right)}} \right)\\ &= P\left({\xi \ge \sum\limits_{j,{d_{{\text{free}}}}} {\left({{{\left| {{\lambda_{l}}\left({{x_{k}^{l}}(I) - \hat{x}_{k}^{l}(I)} \right)} \right|}^{2}} + {{\left| {{\lambda_{f(l)}}\left({{x_{k}^{l}}(Q) - \hat{x}_{k}^{l}(Q)} \right)} \right|}^{2}}} \right)}} \right), \end{aligned}  $$

where $\sum \limits _{j,{d_{{\text {free}}}}}$ denotes the summation of the *d*_free_ terms with index *j*. $\xi = \sum \limits _{j,{d_{{\text {free}}}}} \left [2{\lambda _{l}}\left ({{x_{k}^{l}}(I) - \hat {x}_{k}^{l}(I)} \right){n_{k}^{l}}(I)+2{\lambda _{f(l)}}\left ({{x_{k}^{l}}(Q) - \hat {x}_{k}^{l}(Q)} \right){n_{k}^{l}}(Q) \right ]$. Because of ${n_{k}^{l}}(I)$ and ${n_{k}^{l}}(Q)$ are independent Gaussian random variables with variance ${\sigma ^{2}} = \frac {{{N_{0}}}}{2}$, *ξ* is a Gaussian random variable with zero mean and variance $\sigma _{\xi }^{2} =$$\sum \limits _{i,{d_{{\text {free}}}}} {\left [ {4{{\left | {{\lambda _{l}}\left ({{x_{k}^{l}}(I) - \hat {x}_{k}^{l}(I)} \right)} \right |}^{2}} + 4{{\left | {{\lambda _{f(l)}}\left ({{x_{k}^{l}}(Q) - \hat {x}_{k}^{l}(Q)} \right)} \right |}^{2}}} \right ] \cdot {\sigma ^{2}}}$. (7) can be expressed by the Gaussian Q-function as [8, 45]

(8) $$ \begin{aligned} P\left({{\textbf{c}_{l}} \to \hat{\mathbf{c}}_{l}|\textbf{H}} \right) &\le Q\left({\sqrt {\frac{{\sum\limits_{j,{d_{{\text{free}}}}} {\left({{{\left| {{\lambda_{l}}\left({{x_{k}^{l}}(I) - \hat{x}_{k}^{l}(I)} \right)} \right|}^{2}} + {{\left| {{\lambda_{f(l)}}\left({{x_{k}^{l}}(Q) - \hat{x}_{k}^{l}(Q)} \right)} \right|}^{2}}} \right)} }}{{2{N_{0}}}}}} \right)\\ &\le Q\left({\sqrt {\frac{{{d_{{\text{free}}}}\left({d_{\min,I}^{2}{\lambda_{l}^{2}} + d_{\min,Q}^{2}\lambda_{f(l)}^{2}} \right)}}{{2{N_{0}}}}}} \right), \end{aligned}  $$

where $Q\left (x \right) = \frac {1}{\pi }\int _{0}^{{\pi \left /\right. 2}} {\exp \left (- \frac {x^{2}}{2{{\sin }^{2}}\theta } \right)d\theta }$, (*x*≥0). *d*_min,*I*_ and *d*_min,*Q*_ are minimum Euclidean distance of the I-component and Q-component of two symbols, respectively. For the rotated QAM constellation, it is easy to get that *d*_min,*I*_=*d*_min,*Q*_=*d*_min,*cp*_. Using an upper bound for the Q function $Q\left (x \right) \le \frac {1}{2}\exp \left ({ - \frac {{{x^{2}}}}{2}} \right)$, the the average PEP can be upper bounded as

(9) $$ \begin{aligned} P\left({\textbf{c}_{l}} \to \hat{\mathbf{c}}_{l} \right) &= {{\mathbb{E}}_{\textbf{H}}}\left[ {P\left({\textbf{c}_{l}} \to \hat{\mathbf{c}}_{l}|\textbf{H} \right)} \right]\\ &\le {{\mathbb{E}}_{{\lambda_{l}},{\lambda_{f(l)}}}}\left[ {\frac{1}{2}\exp \left({ - \frac{{{d_{{\text{free}}}}d_{\min,cp}^{2}\left({{\lambda_{l}^{2}} + \lambda_{f(l)}^{2}} \right)}}{{4{N_{0}}}}} \right)} \right]. \end{aligned}  $$

From (9), we can know that the error performance of *l*th layer is related to *λ*_*l*_ and *λ*_*f*(*l*)_. For the spatial multiplexing MIMO system, the performance is dominated by the weakest subchannel. That means the performance of proposed scheme should improve as the smallest ${{\lambda _{l}^{2}} + \lambda _{f(l)}^{2}}$ over all layer increases. In fact, the spatial Q-component interleaved sequence {*λ*_*f*(*l*)_|*l*=1,2,…,*M*} can be viewed as a rearrangement of sequence {*λ*_1_,*λ*_2_,…,*λ*_*M*_}. In order to obtain the best performance, we should find an optimum spatial Q-component interleaved sequence from the full permutation of all elements in sequence {*λ*_1_,*λ*_2_,…,*λ*_*M*_}. The design criterion of optimum spatial Q-component interleaver can be written as

(10) $$ {f_{opt}} = \arg \mathop{\max}\limits_{f} \left[ {\mathop{\min}\limits_{l \in [1,M]} \left({{\lambda_{l}^{2}} + \lambda_{f(l)}^{2}} \right)} \right].  $$

### **Lemma1**.

For the SVD-precoded *M* layer spatial multiplexing MIMO system with spatial Q-component interleaver, the descending-order eigenvalues of MIMO channel matrix are *λ*_1_>*λ*_2_>⋯>*λ*_*M*_. The optimum Q-component interleaving rule is *f*(*l*)=*M*−*l*+1, *l*=1,2,…,*M*, which brings ${\mathop {\min }\limits _{l \in [1,M]} \left ({{\lambda _{l}^{2}} + \lambda _{f(l)}^{2}} \right)}$ to its maximum value.

### *Proof*.

See Appendix. □

From Lemma 1, we prove that the component interleaver used in [40] is optimal for the CISM scheme with SVD precoding.

## Limited feedback precoding for the proposed scheme

The most serious drawback of SVD precoding is the requirement of complete CSI at both the transmitter and receiver. To reduce the data rate requirement for the feedback channel, LF precoding has drawn much attention and has been widely used in practical systems.

For the proposed scheme with LF precoding, the *N*_*T*_×*M* precoding matrix **F** shall be selected in a codebook set ${\mathcal {P}} = \left \{ {{\textbf {F}_{1}},{\textbf {F}_{2}}, \ldots,{\textbf {F}_{{N_{p}}}}} \right \}$. The precoding matrix index (PMI) of the selected precoding matrix shall be sent from the receiver to the transmitter via a reliable feedback channel in time, which informs the transmitter to carry out the corresponding precoding. In the receiver, the linear ZF MIMO detection matrix is

(11) $$ \textbf{G} = {\left[ {{{\left({\textbf{H}_{p}} \right)}^{H}}\left({\textbf{H}_{p}} \right)} \right]^{- 1}}{\left({\textbf{H}_{p}} \right)^{H}},  $$

where **H**_*p*_=**HF**.

For the spatial multiplexing MIMO system without spatial Q-component interleaver, the optimal precoder selection criteria from a codebook were proposed in [13, 14]. It is shown that in order to minimize a bound on the average probability of a symbol vector error, the minimum substream SNR *SNR*_min_ must be maximized. A close approximation to maximizing the minimum SNR for ZF receiver is also provided under the assumption of *M*<*N*_*T*_. These two error rate-based percoding matrix selection criteria (SC) are summarized as follows.

***SC 1 - Maximum minimum SNR***.

(12) $$ \textbf{F} = \mathop{\arg \max }\limits_{{\textbf{F}_{k}},{\textbf{F}_{k}} \in {\mathcal{P}}} \left[{\mathop {\min}\limits_{l \in [1,M]} \left({{SNR}_{l}^{{\textbf{F}_{k}}}} \right)} \right],  $$

where ${{SNR}_{l}^{{\textbf {F}_{k}}}}$ is the SNR for layer *l* corresponding to the precoding matrix **F**_*k*_.

***SC 2 - Maximum minimum singular value***.

(13) $$ \textbf{F} = \mathop{\arg \max }\limits_{{\textbf{F}_{k}},{\textbf{F}_{k}} \in {\mathcal{P}}} \left[ {{\lambda_{\min}}\left({\textbf{H}{\textbf{F}_{k}}} \right)} \right],  $$

where *λ*_min_(**HF**_*k*_) is the minimum singular value of **HF**_*k*_.

However, the selection criteria above are unsuitable for the proposed scheme with ZF receiver because spatial Q-component interleaving readjusts the SNR of each layer. Due to the orthogonal modulation, the transmit powers of the I-component and that of the Q-component on each layer are both $\frac {{{E_{s}}}}{2}$. By the similar methods shown in [47], the effective SNR of the received symbol on the *l*th layer after spatial Q-component deinterleaving can be simplified to

(14) $$ \overline{SNR}_{l}^{{\text{eff}}} = \frac{{2{E_{s}}}}{{{N_{0}}\left({\left[{\textbf{H}_{p}^{H}{\textbf{H}_{p}}} \right]_{ll}^{- 1} + \left[ {\textbf{H}_{p}^{H}{\textbf{H}_{p}}} \right]_{f(l)f(l)}^{- 1}} \right)}},  $$

where $\left [\textbf {A} \right ]_{ll}^{- 1}$ is entry (*l*,*l*) of ***A***^−1^. Assume that the probability of symbol error for the *l*th layer is *P*_*l*_ and ${P_{\max }} = \mathop {\max }\limits _{l \in [1,M]} \left ({{P_{l}}} \right)$. Depending on the input constellation, the performance of the proposed scheme with spatial Q-component interleaving in terms of vector symbol error rate can be computed as [45, 47]

(15) $$ \begin{aligned} {P_{M,ve}} &= 1 - \prod\limits_{l = 1}^{M} {\left({1 - {P_{l}}} \right)} \\ &\le 1 - {\left({1 - {P_{\max }}} \right)^{M}}\\ &\approx M{P_{\max }}\\ &\le M{N_{e}}Q\sqrt {{\overline {SNR}}_{\min }^{{\rm{eff}}}\frac{{d_{\min }^{2}}}{2}}, \end{aligned}  $$

where $\overline {SNR}_{\min }^{{\text {eff}}} = \mathop {\min }\limits _{l \in [1,M]} \left ({{\overline {SNR}}_{l}^{{\text {eff}}}} \right)$. ${d_{\min }^{2}}$ is the squared minimum distance and *N*_*e*_ is the average number of nearest neighbors of the per-layer constellation. Therefore, the performance of the proposed scheme is dominated by the minimum effective SNR of *M* layers. Thus, based on the optimal error performance, the optimal precoding matrix selection criterion that is applicable to the proposed scheme can be expressed as follows.

***SC 3 - Maximum minimum effective SNR***.

(16) $$ \textbf{F} = \mathop {\arg \max}\limits_{{\textbf{F}_{k}},{\textbf{F}_{k}} \in {\mathcal{P}}} \left[ {\mathop {\min}\limits_{l \in \left[ {1,M} \right]} \left({\overline {SNR}_{l}^{{\text{eff}},{\textbf{F}_{k}}}} \right)} \right],  $$

where ${\overline {SNR}_{l}^{{\text {eff}},{\textbf {F}_{k}}}}$ is the effective SNR for layer *l* corresponding to the precoding matrix **F**_*k*_. For each precoding matrix $\textbf {F}_{k} \in {\mathcal {P}}$, the matrix with the largest $\overline {SNR}_{\min }^{{\text {eff}}}$ is chosen.

SC 3 can provide the optimal error performance for the proposed scheme. However, to calculate $\overline {SNR}_{\min }^{{\text {eff}}}$, it requires a search over all layers according to (14) based on the spatial Q-component interleaving rule for all alternative precoding matrix ${\textbf {F}_{k}} \in {\mathcal {P}}$. The computational complexity is much high, especially when *M* and the size of codebook are large. This motivates us to design a novel precoder selection criterion for the proposed scheme based on the error performance with computational reduction.

For the two-layer spatial multiplexing system, the Q-components of symbols on two layers are exchanged with each other by Q-components interleaving. According to (14), the practical SNRs of the received symbols on both layers after spatial Q-component deinterleaving are equal and can be expressed as follows

(17) $$ \overline {SNR}^{{\text{eff}}} = \frac{{2{E_{s}}}}{{{N_{0}}\left({\left[ {\textbf{H}_{p}^{H}{\textbf{H}_{p}}} \right]_{11}^{- 1} + \left[ {\textbf{H}_{p}^{H}{\textbf{H}_{p}}} \right]_{22}^{- 1}} \right)}}.  $$

Correspondingly, the two layers have the same performance. Assume that the probability of symbol error for the two layers are both *P*_*s*_=*P*_1_=*P*_2_. According to (15), the vector symbol error rate of the two-layer proposed scheme with spatial Q-component interleaving can be computed as

(18) $$ {P_{2,ve}} \approx 2{P_{s}} \le 2{N_{e}}Q\left({\sqrt {\overline {SNR}^{{\text{eff}}}\frac{{d_{\min}^{2}}}{2}}} \right).  $$

As shown above, in order to minimize a bound on the average probability of a symbol vector error, $\overline {SNR}^{{\text {eff}}}$ must be maximized. Note that

(19) $$ \begin{aligned} {\rm{tr}}\left({{{\left[ {\textbf{H}_{p}^{H}{\textbf{H}_{p}}} \right]}^{- 1}}} \right) &= \left[ {\textbf{H}_{p}^{H}{\textbf{H}_{p}}} \right]_{11}^{- 1} + \left[{\textbf{H}_{p}^{H}{\textbf{H}_{p}}} \right]_{22}^{- 1}\\ &= \sum\limits_{k = 1}^{2} {{\rho_{k}}\left({{{\left[ {\textbf{H}_{p}^{H}{\textbf{H}_{p}}} \right]}^{- 1}}} \right)} \\ &= \sum\limits_{k = 1}^{2} {\frac{1}{{{\rho_{k}}\left({\left[ {\textbf{H}_{p}^{H}{\textbf{H}_{p}}} \right]} \right)}}} \\ &= \sum\limits_{k = 1}^{2} {\frac{1}{{{\lambda_{k}^{2}}\left({{\textbf{H}_{p}}} \right)}}}, \end{aligned}  $$

where *ρ*_*k*_(**A**) and *λ*_*k*_(**A**) are the *k*th eigenvalue and singular value of **A**, respectively. Therefore, (17) can be simplified as

(20) $$ \overline{SNR}_{l}^{{\text{eff}}} = \frac{{2{E_{s}}}}{{{N_{0}}}}\left({{\sum\limits_{k = 1}^{2} {\frac{1}{{{\lambda_{k}^{2}}\left({{\textbf{H}_{p}}} \right)}}} }} \right)^{-1}.  $$

In order to achieve the maximum $\overline {SNR}^{{\text {eff}}}$, it has to make sure that (19) has the minimum value. Therefore, for the proposed scheme with *M*=2, the criterion that choosing the precoding matrix with the minimum value of $\sum \limits _{k = 1}^{2} {\frac {1}{{{\lambda _{k}^{2}}\left ({{\textbf {H}_{p}}} \right)}}}$ leads to the optimal error performance.

Based on (14) and (19), for the proposed scheme with *M*>2, we can lower-bound $\overline {SNR}_{\min }^{{\text {eff}}}$ as

(21) $$ \begin{aligned} \overline{SNR}_{\min}^{{\text{eff}}} &\ge \frac{{2{E_{s}}}}{{{N_{0}}\left({\sum\limits_{l = 1}^{M} {\left[ {\textbf{H}_{p}^{H}{\textbf{H}_{p}}} \right]_{ll}^{- 1}}} \right)}} = \frac{{2{E_{s}}}}{{{N_{0}}}}\left({{\sum\limits_{l = 1}^{M} {\frac{1}{{{\lambda_{l}^{2}}\left({{\textbf{H}_{p}}} \right)}}} }} \right)^{-1}. \end{aligned}  $$

Through the above analysis, we find a simple method to calculate the minimum effective SNR $\overline {SNR}_{\min }^{{\text {eff}}}$ for SC 3. Instead of the calculation of effective SNR for each layer that is related to ${\left [ {\textbf {H}_{p}^{H}{\textbf {H}_{p}}} \right ]^{- 1}}$, the simplified method only need to compute the singular value of **H**_*p*_. We define that $g\left ({{\textbf {F}_{k}}} \right) \buildrel \Delta \over = \sum \limits _{l = 1}^{M} {\frac {1}{{{\lambda _{l}^{2}}\left (\textbf {HF}_{k} \right)}}}$. A precoding matrix selection criterion with low complexity can be expressed as follows.

***SC 4 - Minimum singular value function***.

(22) $$ \textbf{F} = \mathop{\arg \min}\limits_{{\textbf{F}_{k}},{\textbf{F}_{k}} \in {\mathcal{P}}} \left[ {g\left({{\textbf{F}_{k}}} \right)} \right]  $$

For each precoding matrix $\textbf {F}_{k} \in {\mathcal {P}}$, the matrix with the minimum *g*(**F**_*k*_) is chosen.

It is worth noting that for the proposed scheme with *M*=2, SC 4 are equal to SC 3.

## Optimal rotation angle based on AMI analysis

### BICM-AMI and motivation of optimization

In the practical communication system, the transmitted signal ${\mathbf {x}}={\left [{x_{k}^{1}},{x_{k}^{2}},\ldots,{x_{k}^{M}}\right ]^{T}}$ usually takes on a discrete finite alphabet (constellation signal set). Assuming equiprobable inputs, the AMI between the signal after rotated constellation mapping and the signal after the soft demapper is named BICM-AMI [1].

According to Lemma 1 in [32], an effective way to calculate the BICM-AMI by measuring the AMI between the transmitted bits and their corresponding LLRs can be expressed as

(23) $$ \begin{aligned} {I_{{\text{BICM}}}} &= \sum\limits_{l = 1}^{M} {\sum\limits_{j = 1}^{m} {I\left({{c_{l}^{j}};L_{{{c_{l}^{j}}}}} \right)}} \\ &= Mm + \sum\limits_{l = 1}^{M} {{{\mathbb{E}}_{{L_{{c_{l}^{j}}}}}}\left[ {{g_{I}}\left({{L_{{c_{l}^{j}}}}} \right)} \right]}, \end{aligned}  $$

where ${g_{I}}\left (x \right) = \frac {{{e^{x}}}}{{1 + {e^{x}}}}{\log _{2}}\frac {{{e^{x}}}}{{1 + {e^{x}}}} + \frac {1}{{1 + {e^{x}}}}{\log _{2}}\frac {1}{{1 + {e^{x}}}}$.

For the proposed scheme, the optimal constellation rotation angle is one of the most important problems for SSD. For the coded MIMO system, a number of factors (the number of antennas, precoding, modulation, code rate, and so on) will impact the optimal angle. As a result, the selection of the optimum angle becomes much more challenging. Maximizing BICM-AMI is an effective way to optimize the system performance. In [43], the BICM-AMI is used to design the optimal rotation angle of trace-orthonormal STBC by establishing the relationship between the optimal angle and SNR. It inspires us to optimize the rotation angle of the proposed scheme in the same way. In this paper, BICM-AMI is first used to evaluate the performance advantages of the optimal component interleaver and precoding matrix selection criterion proposed in Sections 3 and 4. Based on AMI maximization criterion, determination of the optimal rotation angle by establishing the relationship curve between optimal angle and corresponding AMI is investigated.

### BICM-AMI of the proposed schemes

**Fig. 2:**
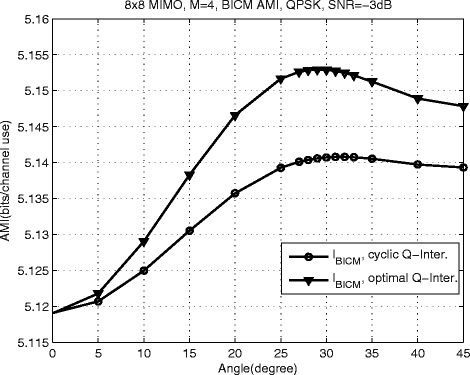
*N*_*R*_=*N*_*T*_=8, *M*=4 SVD-precoded MIMO system, *I*_BICM_ vs. *θ* for QPSK at SNR=-2.6 dB

**Fig. 3:**
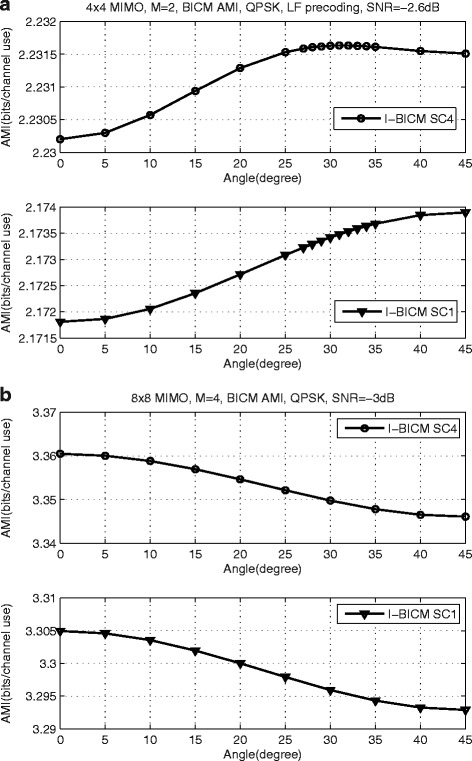
LF precoded MIMO system, *I*_BICM_ vs. *θ* for QPSK. **a** *N*_*R*_=*N*_*T*_=4, *M*=2, SNR=-2.6 dB. **b** *N*_*R*_=*N*_*T*_=8, *M*=4, SNR=-3 dB

The AMI reflects the maximum information rate that can be reliably transmitted at a given SNR. Therefore, The larger AMI value indicates a better system performance. For a given SNR, the BICM-AMI value *I*_*BICM*_ varies with the rotation angle. Some examples are presented in Figs. 2 and 3. Without loss of generality, for the proposed scheme with LF precoding, the precoding codebooks for *N*_*R*_=*N*_*T*_=4 and *N*_*R*_=*N*_*T*_=8 MIMO schemes are based on [5, 48], respectively.

For the proposed MIMO system based on SVD precoding with *N*_*R*_=*N*_*T*_=8, *M*=4, in order to verify the performance advantage of the optimal Q-component interleaver, the commonly used cyclic Q-component interleaver is also investigated for comparison purpose, which can be expressed as

(24) $$ f(l)=(l~\text{mod}~ M)+1, l=1,2,\ldots,M.  $$

The numeric results are shown in Fig. 2. The optimum rotation angle for SVD-precoded MIMO system with optimal Q-component interleaver is 29°, while it is 32° for the proposed scheme with cyclic Q-component interleaver. It is worth noting that the AMI of the proposed scheme with optimum Q-component interleaver is larger than that of the proposed scheme with cyclic Q-component interleaver. This observation means that the optimum Q-component interleaver proposed in Lemma I outperforms the commonly used cyclic Q-component interleaver.

For the LF precoded MIMO system, the AMIs of proposed systems with SC 1 and SC 4 are plotted in the same figure. As can be seen from the Fig. 3a, for both percoding matrix selection criteria, the optimal rotation angles of the proposed MIMO system with *N*_*R*_=*N*_*T*_=4, *M*=2 are different at SNR = −2.6 dB, which are about 45° and 31° for SC 1 and SC 4, respectively. For *N*_*R*_=*N*_*T*_=8, *M*=4 MIMO system based on LF precoding with the cyclic Q-component interleaver at SNR = −3 dB, the optimal angles for SC 1 and SC 4 are both 0°. From Fig. 3, it can be observed that BICM-AMI for SC 4 is always not less than that of SC 1. This observation indicates that the proposed MIMO scheme with SC 4 has a better performance than the proposed scheme with SC 1.

**Fig. 4:**
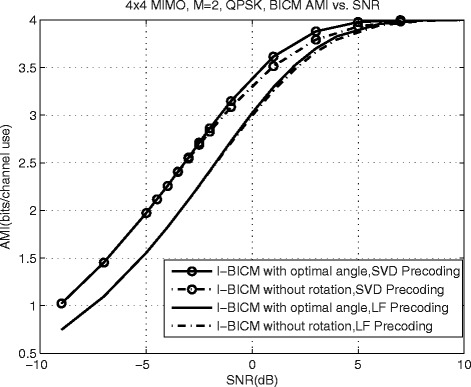
BICM-AMI corresponding to the proposed schemes with optimal rotation angles and without rotation for *N*_*R*_=*N*_*T*_=4, *M*=2 MIMO

For the purpose of studying the effect of constellation rotation on the system performance, for the proposed *N*_*R*_=*N*_*T*_=4, *M*=2 MIMO scheme, the BICM AMIs corresponding to the proposed schemes with optimal rotation angles and without rotation are plotted in Fig. 4. For the proposed schemes with SVD and LF precoding, constellation rotation brings an increase of AMI. However, for the proposed schemes with LF precoding, the BICM-AMI increased by rotation is slight. It means that compared with SVD-precoded scheme, the performance improvement of the LF precoded proposed schemes caused by constellation rotation is very limited.

### Determination method of optimal rotation angle

**Fig. 5:**
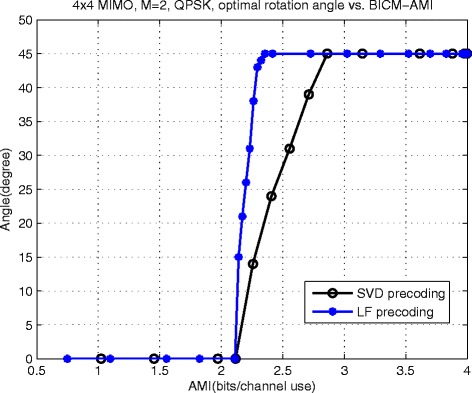
*θ* ^opt^ vs. *I*_BICM_(*S* *N* *R*,*θ* ^opt^) for *N*_*R*_=*N*_*T*_=4, *M*=2 MIMO

By the same method introduced in [43], based on BICM-AMI maximization criterion, the optimal rotation angle *θ*^opt^ and corresponding *I*_BICM_(*SNR*,*θ*^opt^) can be obtained at a given SNR. We can get the relationship between *I*_BICM_(*SNR*,*θ*^opt^) and *θ*^opt^ by making various adjustments of SNR. For *N*_*R*_=*N*_*T*_=4, *M*=2 MIMO system, the the curve of *θ*^opt^ versus *I*_BICM_(*SNR*,*θ*^opt^) is shown in Fig. 5.

From Fig. 5, the optimal angle *θ*^opt^ can be viewed as a function of *I*_BICM_, i.e., *θ*^opt^=*κ*(*I*_BICM_). It provides a good reference for selecting the optimal angle. For the proposed *M*-layer BICM MIMO system with code rate *R* and 2^*m*^-ary modulation, the theoretical maximum information rate that can be transmitted is ${\bar {I}_{\max }} = MmR$. Therefore, the optimal rotation angle is highly recommended to choose ${\theta ^{{\text {opt}}}} = \kappa \left ({{\bar {I}_{\max }}} \right)$. For instance, for *N*_*R*_=*N*_*T*_=4, *M*=2 MIMO system with 0.5 code rate and QPSK modulation, ${\bar {I}_{\max }} = 2$. According to Fig. 5, the optimal rotation angle are both 0° for SVD-precoded proposed scheme and LF precoded proposed scheme. According to the above method, we can get the optimal rotation angles for proposed scheme based on various parameters without predetermined operating SNR. Because of limitations of space, the optimal rotation angles that are used in Section 6 are listed in Table 1.

**Table 1:** The optimal rotation angles for the proposed schemes with Gray labeled QPSK

*N*_*R*_	*N*_*T*_	*M*	*R*	Modulation	Precoding	*θ* ^opt^
8	8	4	3/4	QPSK	SVD	45°
8	8	6	3/4	QPSK	SVD	27°
8	8	8	3/4	QPSK	SVD	25°
4	4	2	1/2	QPSK	LF SC 4	0°
4	4	2	3/4	QPSK	LF SC 4	45°
8	8	2, 4, 6	1/2	QPSK	LF SC 4	0°

## Simulation result

In this section, simulation results are provided to illustrate the performance of the proposed spatial multiplexing MIMO system with spatial component interleaver. In the simulation, 1/2-rate 64-state BCC with the generator of (133,171)_8_ is used as the channel code. The high code rate *R*=3/4 is derived from it by employing “puncturing” as introduced in [49]. The coded bit length *N*=1200. The decoding of BCC is the standard Bahl-Cocke-Jelinek-Raviv (BCJR) algorithm. The i.i.d. Rayleigh MIMO fading channel is employed and the channel is changed independently from each block of *N* coded bits.

### Results of SVD-precoded scheme

**Fig. 6:**
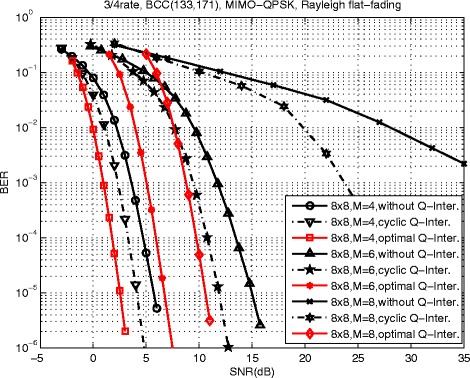
BER performance of 3/4-rate BCC coded, QPSK MIMO systems with SVD precoding, *N*_*R*_=*N*_*T*_=8

In order to verify the effectiveness of the proposed optimum spatial Q-component interleaver, the BER performance compared with commonly used cyclic Q-component interleaver proposed in (24) is presented for *N*_*R*_=*N*_*T*_=8 3/4-rate BCC-coded MIMO systems. It is observed in Fig. 6 that the proposed scheme with cyclic Q-component interleaver provides a significant performance improvement with respect to the conventional spatial multiplexing MIMO scheme. Furthermore, the proposed scheme with optimum spatial Q-component interleaver proposed in Lemma I can still obtain about 17 dB for *M*=8, 4.8 dB for *M*=6 and 1.5 dB for *M*=4 at BER = 10^−4^ than the proposed scheme with cyclic Q-component interleaver. From the simulation results, an observation can be made that the SNR gains are greater for more layers.

### Results of LF precoded scheme

**Fig. 7:**
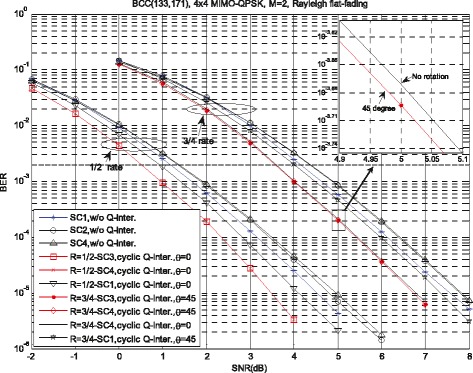
BER performance of BCC coded, QPSK MIMO systems with LF precoding, *N*_*R*_=*N*_*T*_=4, *M*=2

For the proposed scheme with LF precoding and ZF receiver, simulations are carried out to compare the performance of different percoding matrix selection criteria. Figure 7 presents the BER performance for 1/2-rate and 3/4-rate BCC coded *N*_*R*_=*N*_*T*_=4, *M*=2 MIMO systems with 3GPP LTE precoding codebook [5]. Without loss of generality, the commonly used cyclic Q-component interleaver is used. It is clearly shown in Fig. 7 that the proposed scheme with spatial Q-component interleaver always has a performance gain compared to the spatial multiplexing MIMO scheme without Q-component interleaver. For example, for the both schemes with SC 1, the proposed scheme with spatial Q-component interleaver achieves about 0.35 and 0.17 dB SNR gain compared with the spatial multiplexing MIMO scheme without Q-component interleaver at BER = 10^−4^. For the conventional spatial multiplexing MIMO scheme without Q-component interleaver, SC 1 has the best performance and achieves about 0.27 dB SNR gain compared with SC 2 for both code rate at BER = 10^−4^. In contrast, the performance of proposed scheme with SC 1 is worse than that with SC 3 and SC4 about 0.5 dB. This observation indicates that the proposed SC 3 and SC 4 are more suitable for the proposed LF MIMO system with spatial Q-component interleaver. For the 3/4-rate BCC-coded proposed scheme, the optimal rotation angle is 45°. However, the performance improvement caused by rotation is very limited, which is consistent with the AMI analysis in Fig. 4. It is worth noting that the low complexity precoding matrix selection criterion SC 4 has the same performance with SC 3 for the proposed scheme with *M*=2 and just has a little performance loss compared with SC 2 for the conventional spatial multiplexing MIMO scheme without Q-component interleaver.

**Fig. 8:**
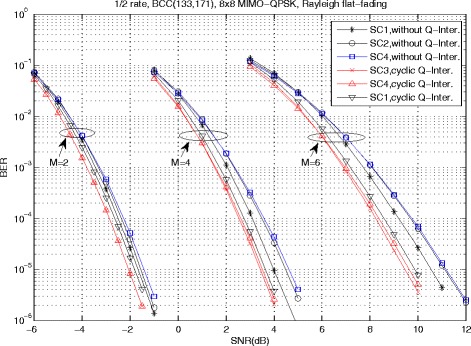
BER performance of 1/2-rate BCC-coded, QPSK MIMO systems with LF precoding, *N*_*R*_=*N*_*T*_=8

For the MIMO system with *M*>2, the proposed LF precoded schemes with the proposed criteria SC 3 and SC4 always have the effective SNR gains. Figure 8 presents the BER performance for 1/2-rate BCC-coded *N*_*R*_=*N*_*T*_=8 MIMO systems with precoding codebook introduced in [48]. Compared with the conventional spatial multiplexing MIMO scheme with SC 1, the proposed scheme with SC 3 obtains about 0.36, 0.56, and 0.95 dB for *M*=2, *M*=4, and *M*=6, respectively. For *M*=2, the proposed scheme with SC 3 and SC 4 have the same performance, while the performance of SC 4 is about 0.15 dB worse than that of SC 3 for *M*>2. The simulation results illustrate the effectiveness of the proposed system.

## Conclusions

In this paper, precoded BICM spatial multiplexing MIMO system with spatial component interleaver is discussed. For the SVD-precoded spatial multiplexing MIMO system with spatial component interleaver, the PEP of coded bits is analyzed. Based on the bound of PEP, the optimum spatial Q-component interleaver design criterion and a optimum spatial Q-component interleaving rule are proposed. The performance of conventional spatial multiplexing MIMO system is dominated by the smallest singular value of the channel matrix (the weakest layer), while the proposed scheme can improve the performance of the weakest layer through the spatial component interleaving. In addition, the LF precoded BICM spatial multiplexing MIMO system with spatial component interleaver and ZF receiver is studied. Based on the minimum average probability of symbol vector error criteria, the optimal effective SNR-based precoding matrix selection criterion and a simplified criterion are proposed. The simplified criterion has the same performance for two-layer transmission and small performance loss for *M*>2. Moreover, the method of determining the optimal rotation angles for the proposed schemes via maximizing the BICM AMI is also presented. The AMI analysis also illustrates that the optimal rotation angle is more crucial for SVD-precoded MIMO scheme with spatial component interleaver. For the proposed scheme with LF precoding, performance gain brought by constellation rotation is very limited. The simulation results validate all the theoretical analyses. Through the above optimization, the performance of the proposed precoded BICM spatial multiplexing MIMO system with spatial component interleaver outperforms that of conventional precoded spatial multiplexing MIMO system.

## Appendix

### Proof of Lemma 1

Assume that *λ*_*i*_^2^=*ρ*_*i*_ is the *i*th (*i*∈[1,*M*]) largest eigenvalue of **H**·**H**^*H*^. Correspondingly, *ρ*_1_>*ρ*_2_>⋯>*ρ*_*M*_. According to Eq. (10), the optimum spatial Q-component interleaving rule should guarantee that $\eta _{f} ={\mathop {\min }\limits _{l \in [1,M]} \left ({\rho _{l} + \rho _{f(l)}} \right)}$ reaches its maximum value. Note that the interleaved sequence {*ρ*_*f*(1)_,*ρ*_*f*(2)_,…,*ρ*_*f*(*M*)_} is another new permutation of {*ρ*_1_,*ρ*_2_,…,*ρ*_*M*_}. The interleaving rule of proposed optimum spatial Q-component interleaver in Lemma 1 can be list in Table 2.

**Table 2:** Eigenvalue sequences for proposed optimum spatial Q-component interleaver in Lemma 1

***ρ***_*l*_	*ρ*_1_	*ρ*_2_	⋯	*ρ*_*i*−1_	*ρ*_*i*_	*ρ*_*i*+1_	⋯	*ρ*_*M*−1_	*ρ*_*M*_
***ρ***_*f*(*l*)_	*ρ*_*M*_	*ρ*_*M*−1_	⋯	*ρ*_*M*−*i*+2_	*ρ*_*M*−*i*+1_	*ρ*_*M*−*i*_	⋯	*ρ*_2_	*ρ*_1_

From Table 2, the sum of *n*th ($n \in \left [ {1,\left \lfloor {\frac {M}{2}} \right \rfloor } \right ]$) pair of eigenvalues (the *n*th column) is equal to that of (*M*−*n*+1)th pair of eigenvalues (the (*M*−*n*+1)th column). Without loss of generality, we assume that the sum of the *i*th ($1 \le i \le \left \lfloor {\frac {M}{2}} \right \rfloor $) pair of eigenvalues has the minimum value, that is *η*_*f*_=*ρ*_*i*_+*ρ*_*M*−*i*+1_.

Suppose Lemma 1 is not tenable, we can find a new interleaved sequence corresponding to the new interleaving rule *f*^′^, that has ${{\eta }_{{f}^{\prime }}} > {\eta _{f}}$. In order to obtain a larger minimum value, for the new rule *f*^′^, *ρ*_*f*(*i*)_=*ρ*_*M*−*i*+1_ that corresponding to the minimum value *η*_*f*_ must be changed. In fact, the changing of *ρ*_*f*(*i*)_ can be viewed as exchanging position with another eigenvalue *ρ*_*k*_, (*k*≠*M*−*i*+1). Correspondingly, *f*^′^(*i*)=*k*, and *f*^′^(*f*(*k*))=*M*−*i*+1. The eigenvalue sequences for new spatial Q-component interleaver rule *f*^′^ is listed in Table 3.

**Table 3:** Eigenvalue sequences for new spatial Q-component interleaver rule *f* ^′^

***ρ***_*l*_	⋯	*ρ*_*i*_	⋯	*ρ*_*M*−*k*+1_	⋯
$\boldsymbol {\rho }_{f^{\prime }(l)}\phantom {\dot {i}\!}$	⋯	*ρ*_*k*_	⋯	*ρ*_*M*−*i*+1_	⋯

In the case of *k*>*M*−*i*+1, we can get ${\eta _{{f}^{\prime }}} \le {\rho _{i}} + {\rho _{k}} < {\rho _{i}} + {\rho _{M - i + 1}}\phantom {\dot {i}\!}$. Therefore, it always has ${\eta _{{f}^{\prime }}}<{\eta _{f}}\phantom {\dot {i}\!}$.

If *k*<*M*−*i*+1, *M*−*k*+1>*i*. For the new spatial Q-component interleaver rule *f*^′^, ${\rho _{i}} + {\rho _{k}} > {\rho _{M - k + 1}} + {\rho _{M - i + 1}} \ge {\eta _{{f}^{\prime }}}\phantom {\dot {i}\!}$. Because ${\rho _{i}} + {\rho _{M - i + 1}} > {\rho _{M - k + 1}} + {\rho _{M - i + 1}} \ge {\eta _{{f}^{\prime }}}\phantom {\dot {i}\!}$, it always has ${\eta _{{f}^{\prime }}}<{\eta _{f}}\phantom {\dot {i}\!}$.

In a word, we cannot find a new spatial Q-component interleaver rule *f*^′^ which has ${\eta _{{f}^{\prime }}} > {\eta _{f}}\phantom {\dot {i}\!}$. *f*(*l*)=*M*−*l*+1 is the optimum Q-component interleaving rule to assure the maximum value of ${\mathop {\min }\limits _{l \in [1,M]} \left ({{\lambda _{l}^{2}} + \lambda _{f(l)}^{2}} \right)}$.

## References

[1] Caire G, Taricco G, Biglieri E (1998). Bit-interleaved coded modulation. IEEE Trans. Inf. Theory.

[2] Martinez A, Guillen i Fabregas A, Caire G (2006). Error probability analysis of bit-interleaved coded modulation. IEEE Trans. Inf. Theory.

[3] Vucetic B, Yuan J (2003). *Space-Time Coding*.

[4] IEEE Std. P802.11ac/D6.0: Part 11: Wireless LAN medium access control (MAC) and physical layer (PHY) specifications. Amend. 4: Enhancements for Very High Throughput for Operation sin Bands Below 6 GHz (2013).

[5] 3rd Generation Partnership Project; Technical specification group radio access network; Evolved universal terrestrial radio access (E-UTRA); Physical channels and modulation (release 12). 3GPP TS 36.211, version 12.2.0 (2014). http://www.3gpp.org/ftp/Specs/archive/36_series/36.211/.

[6] Telatar E (1999). Capacity of multi-antenna gaussian channels. Eur. Trans. Telecommun..

[7] Foschini GJ (1996). Layered space-time architecture for wireless communication in fading environment when using multiple antennas. Bell Labs. Techn. J..

[8] Goldsmith A (2004). *Wireless Communications*.

[9] Love D, Heath R, Strohmer T (2003). Grassmannian beamforming for multiple-input multiple-output wireless systems. IEEE Trans. Inf. Theory.

[10] Akay E, Sengul E, Ayanoglu E (2007). Bit interleaved coded multiple beamforming. IEEE Trans. Commun..

[11] Sengul E, Hong J, Ayanoglu E (2009). Bit-interleaved coded multiple beamforming with imperfect CSIT. IEEE Trans. Commun..

[12] Boyu L, Ayanoglu E (2012). Multiple beamforming with perfect coding. IEEE Trans. Commun..

[13] Love D, Heath R, Lau V, Gesbert D, Rao B, Andrews M (2008). An overview of limited feedback in wireless communication systems. IEEE J. Selected Areas Commun..

[14] Love D, Heath R (2005). Limited feedback unitary precoding for spatial multiplexing systems. IEEE Trans. Inf. Theory.

[15] Wang H, Li L, Zhang Y, Juntti M (2012). Unitary precoder design for mimo spatial multiplexing systems with limited feedback. *Proceedings of the 2012 IEEE Consumer Communications and Networking Conference (CCNC): 14-17 Jan. 2012*.

[16] Ding L, Liu R, Jiang B, Gao X (2010). Limited feedback unitary precoding using improved euclidean distance metrics for spatial multiplexing systems. *Proceedings of 2010 International Conference on Wireless Communications and Signal Processing (WCSP): 21-23 Oct. 2010*.

[17] Cheng P, Chen Z, Rui Y, Guo Y (2014). Limited feedback unitary precoding for mimo full stream transmission. IEEE Trans. Veh. Technol..

[18] Zheng L, Tse DN (2003). Diversity and multiplexing: a fundamental tradeoff in multiple antenna channels. IEEE Trans. Inf. Theory.

[19] Gamal H, Caire G, Damon M (2004). Lattice coding and decoding achieve the optimal diversity-multiplexing tradeoff of mimo channels. IEEE Trans. Inf. Theory.

[20] Sfar S, Dai L, Letaief KB (2005). Optimal diversity-multiplexing tradeoff with group detection for mimo systems. IEEE Trans. Commun..

[21] E Sengul, E Akay, E Ayanoglu, in *Proceedings of IEEE 61st Vehicular Technology Conference (VTC): 30 May-1 June 2005*. Diversity analysis of single and multiple beamforming (IEEE, 2005), pp. 1293–1296. http://ieeexplore.ieee.org/xpl/articleDetails.jsp?arnumber=1543517&newsearch=true&queryText=Diversity\%20analysis\%20of\%20single\%20and\%20multiple\%20beamforming.

[22] Sfar S, Dai L, Letaief KB (2006). Diversity analysis of single and multiple beamforming. IEEE Trans. Commun..

[23] Tonello AM (2000). Space-time bit-interleaved coded modulation with an iterative decoding strategy. *Proceedings of IEEE 52nd Vehicular Technology Conference (VTC-Fall): 24-28 Sep. 2000*.

[24] Gresset N, Brunel L, Boutros JJ (2008). Space-time coding techniques with bit-interleaved coded modulations for MIMO block-fading channels. IEEE Trans. Inf. Theory.

[25] Ju Park Hong, Ayanoglu E (2010). Diversity analysis of bit-interleaved coded multiple beamforming. IEEE Trans. Commun..

[26] Boutros J, Viterbo E (1998). Signal space diversity: a power and bandwidth efficient diversity technique for the rayleigh fadin channel. IEEE Trans. Inf. Theory.

[27] Kiyani NF, Rizvi UH, Weber JH, Janssen GJM (2007). Optimized rotations for ldpc-coded mpsk constellations with signal space diversity. *Proceedings of IEEE Wireless Communications and Networking Conference: 11-15 March 2007*.

[28] Tran NH, Nguyen HH, Le-Ngoc T (2007). Performance of BICM-ID with signal space diversity. IEEE Trans. Wireless Commun..

[29] Kiyani NF, Weber JH (2007). OFDM with BICM-ID and rotated MPSK constellations and signal space diversity. *Proceedings of IEEE Symposium on Communications and Vehicular Technology in the Benelux: 15-15 Nov. 2007*.

[30] Kiyani NF, Weber JH (2008). EXIT chart analysis of iterative demodulation and decoding of mpsk constellations with signal space diversity. J. Commun..

[31] Zhenzhou M, Zhiping S, Chong Z, Zhongpei Z (2008). Design of signal space diversity based on non-binary ldpc code. *Proceedings of International Conference on Communications, Circuits and Systems: 25-27 May 2008*.

[32] Xie Q, Song J, Peng K, Yang F, Wang Z (2011). Coded modulation with signal space diversity. IEEE Trans. Wireless Commun..

[33] Wu Z, Peng M, Wang W (2012). Improved coding-rotated-modulation orthogonal frequency division multiplexing system. IET Commun..

[34] Yueqian L, Salehi M (2012). Coded MIMO systems with modulation diversity for block-fading channels. *Proceedings of 46th Annual Conference on Information Sciences and Systems (CISS): 21-23 March 2012*.

[35] Heunchul L, Paulraj A (2010). MIMO systems based on modulation diversity. IEEE Trans. Wireless Commun..

[36] Hwang SU, Choi J, Jeon S, Ryu HJ (2009). Performance evaluation of MIMO-OFDM with signal space diversity over frequency selective channels. *Proceedings of IEEE International Symposium on Broadband Multimedia Systems and Broadcasting: 13-15 May 2009*.

[37] S Hong, J Choi, SU Hwang, S Jeon, J-S Seo, in *Proceedings of IEEE 69th Vehicular Technology Conference (VTC): 26-29 April 2009*, ed. by IEEE. Interleaved spatial diversity transmission with coordinate interleaver for MIMO-OFDM systems (IEEE, Barcelona, 2009), pp. 1–4. Weblink: http://ieeexplore.ieee.org/xpl/articleDetails.jsp?arnumber=5073614&newsearch=true&queryText=Interleaved\%20spatial\%20diversity\%20transmission\%20with\%20coordinate\%20interleaver\%20for\%20MIMO-OFDM\%20systems.

[38] Gao X, Wu Z (2012). Joint coding and modulation diversity mimo DFT-S-OFDM scheme. *Proceedings of IEEE 14th International Conference on Communication Technology (ICCT): 9-11 Nov. 2012*.

[39] Wu Z, Gao X (2013). An improved MIMO-OFDM scheme for the next generation WLAN. J. Syst. Eng. Electron..

[40] Srinivas KV, Koilpillai RD, Bhashyam S, Giridhar K (2009). Co-ordinate interleaved spatial multiplexing with channel state information. IEEE Trans. Wireless Commun..

[41] Hong Sungnam, Sagong Min, Lim Chiwoo, Cheun Kyungwhoon (2013). FQAM: a modulation scheme for beyond 4G cellular wireless communication systems. *Proceedings of IEEE Globecom Workshops: 9-13 Dec. 2013*.

[42] Barbieri A, Fertonani D, Colavolpe G (2009). Time-frequency packing for linear modulations: spectral efficiency and practical detection schemes. IEEE Trans. Commun..

[43] El Falou A, Langlais C, Nour CA, Douillard C (2012). Adaptive trace-orthonormal STBC for MIMO system with capacity approaching FEC codes. *Proceedings of IEEE Vehicular Technology Conference (VTC Fall): 3-6 Sept. 2012*.

[44] Goff SY (2003). Signal constellation for bit-interleaved coded modulation. IEEE Trans. Inf. Theory.

[45] Proakis JG (2008). *Digital Communications*.

[46] Park HJ, Ayanoglu E (2009). Diversity analysis of bit-interleaved coded multiple beamforming. *Proceedings of IEEE International Conference on Communications (ICC): 14-18 June 2009*.

[47] Heath RW, Sandhu S, Paulraj A (2001). Antenna selection for spatial multiplexing systems with linear receivers. IEEE Commun. Lett..

[48] R, 1-091246, codebook design for 8 tx transmission in lte-a3GPP TSG RAN WG1 Meeting #56bis. Seoul, Korea (2009). http://www.3gpp.org/ftp/tsg_ran/WG1_RL1/TSGR1_56b/Docs/.

[49] IEEE 802.11n 2009. Part 11: Wireless LAN medium access control (MAC) and physical layer (PHY) specifications, Amendment 5: Enhancements for higher throughput (2009).

